# Supplementary material for: A Zebrafish-Based Platform for High-Throughput Epilepsy Modeling and Drug Screening in F0
Source: Int J Mol Sci. 2024 Mar 4;25(5):2991. doi: 10.3390/ijms25052991 (PMC10931767; doi:10.3390/ijms25052991)
Supplement: Supplementary file 1 [file ijms-25-02991-s001.zip › ijms-2756823-supplementary.pdf]

International Journal of *Molecular Sciences*

1. Supplementary Fgure S1

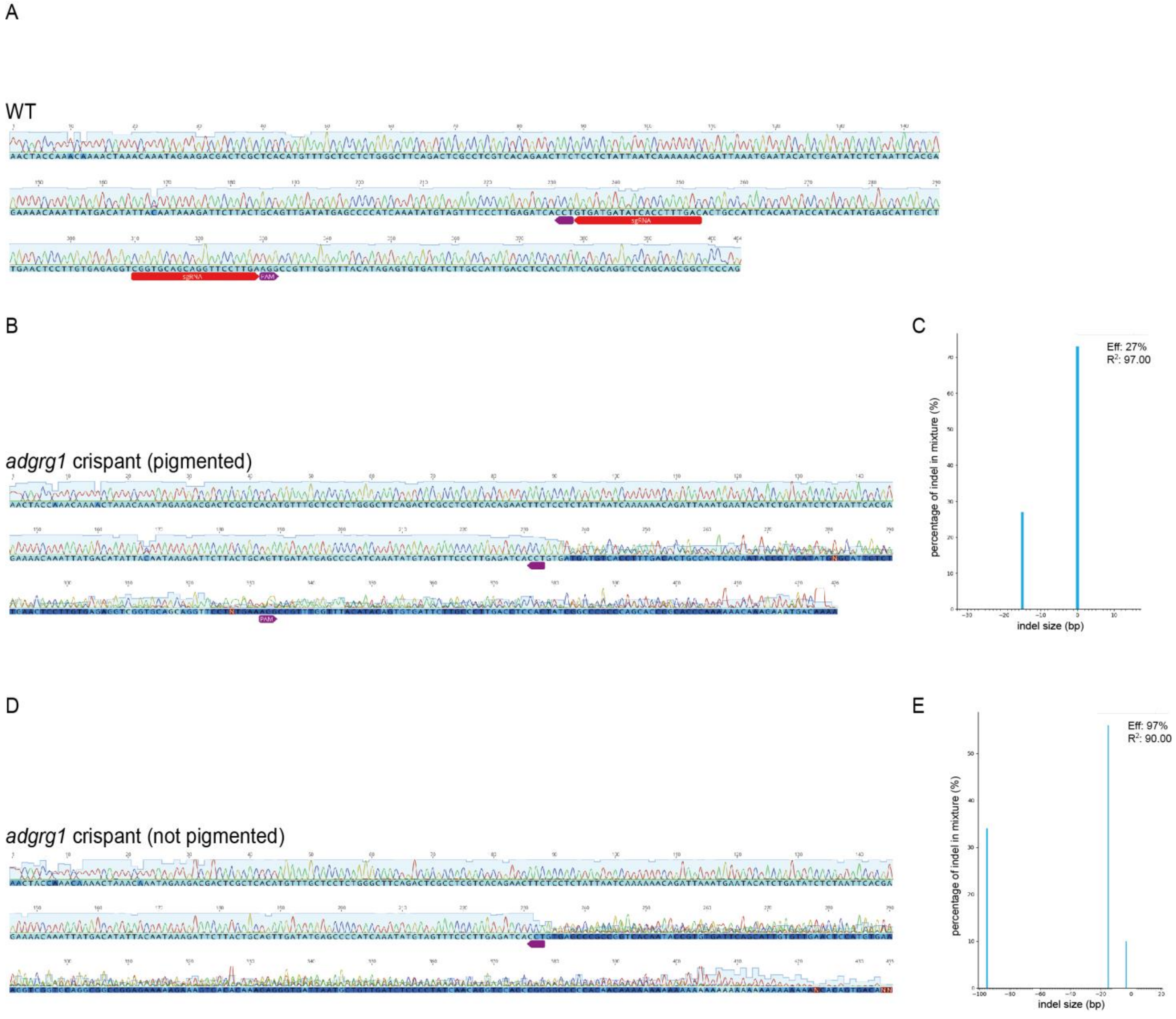

Supplementary Fgure S1. *Tyr* loss-of-function can can be used as an indicator of Cas9 cutting effectiveness. A. Representation of the sequencing results of a wildtype zebrafish larva. The sgRNA used for *adgrg1* crispants generation are represented in orange. The PAM (protospacer adjacent motif) for Cas9 targeting (NGG) is represented in purple. B. Representation of the one of the sequences result of the sequencing of an *adgrg1* crispant with pigmentation. The sgRNA sequences are not present, indicating the proper cleavage of the Cas9. C. Bar graph representing the number of sequences and their percentage of indel described. Only two different sequences were found, being > 70% of them a wildtype sequence. The other sequence had a deletion of >10bp. D. Representation of the one of the sequences result of the sequencing of an *adgrg1* crispant without pigmentation. The sgRNA sequences are not present, as well as one of the PAMs, indicating the proper cleavage of the Cas9. E. Bar graph representing the number of sequences and their percentage of indel described. Three different sequences were found: the wildtype sequence represents <10% of the total amount. Two other sequences with deletions of >10bp and >90bp represent >90% of the sequences.

2. Supplementary Fgure S2

A

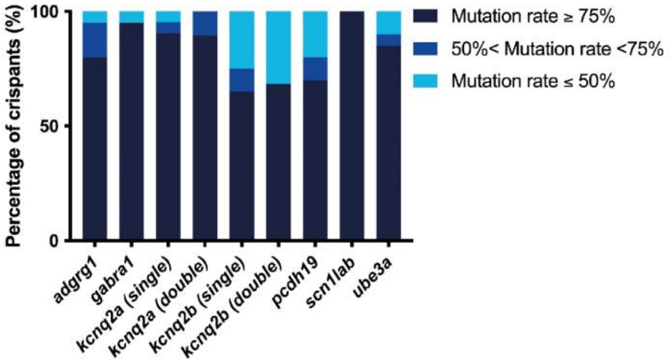

B

	Mutation rate ≥ 75%	50%<Mutation rate<75%	Mutation rate ≤ 50%
adgrg1	80,0	15,0	5,0
gabra1	95,0	0,0	5,0
kcnq2a (single)	89,5	10,5	0,0
kcnq2a (double)	90,5	4,8	4,8
kcnq2b (single)	68,4	0,0	31,6
kcnq2b (double)	65,0	10,0	25,0
pcdh19	70,0	10,0	20,0
scn1lab	100,0	0,0	0,0
ube3a	85,0	5,0	10,0

Supplementary Fgure S2. *Tyr* loss-of-function as a marker for Cas9 cleavage is maintained in all the childhood epilepsy crispants. A. Representation of the analysis of the percentage of the different crispants in three different groups depending on their mutation rate: Mutation rate ≥75% (dark blue), 50% < Mutation rate < 75% (blue) and Mutation rate ≤50% (light blue). B. Table with the values represented in panel A, with all the different studied crispants and the same three groups defined depending on the mutation rate of the injected crispants.

3. Supplementary Fgure S3

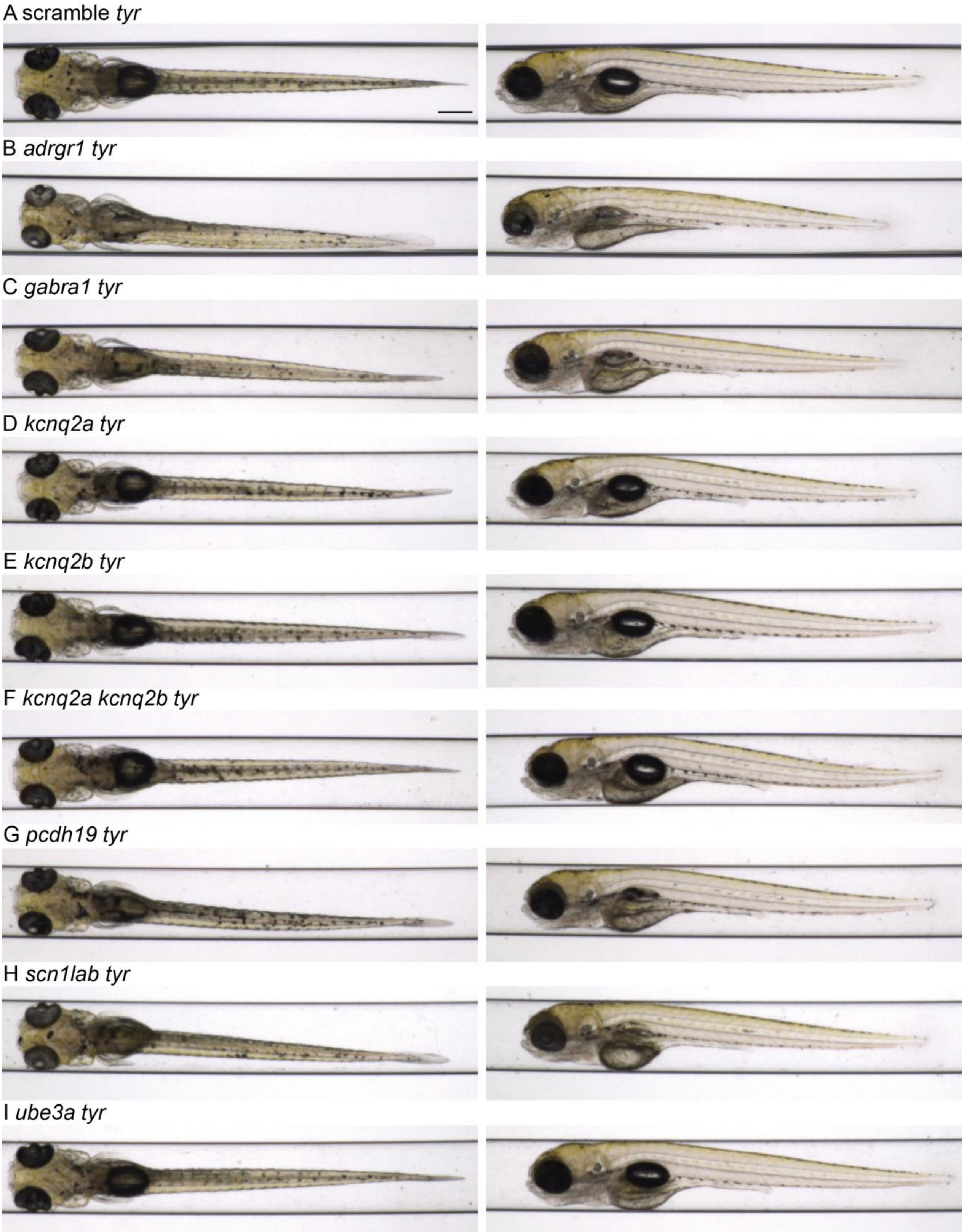

Supplementary Fgure S3. Analysis of morphological phenotypes in the studied epilepsy-associated genes. A,B,C,D,E,F,G,H,I. Representative images of the different crispants injected. The different represented images correspond to scrambled *tyr* (A), *adgrg1 tyr* (B), *gabra1 tyr* (C), *kcnq2a tyr* (D), *kcnq2b tyr* (E), double *kcnq2a kcnq2b tyr* (F), *pcdh19 tyr* (G) *scn1lab tyr* (H) and *ube3a tyr* (I).

4. Supplementary Table S1

		**Body curvature**	**Snout jaw defects**	**Yolk edema**	**Necrosis**	**Tail bending**	**Notochord defects**	**Cranio facial edema**	**Fin absence**	**Scoliosis**
Scrambled (plate A)	Number positive	0	0	3	1	2	0	0	0	0
	Number negative	25	25	25	25	25	25	25	25	25
	% positive	0	0	12	4	8	0	0	0	0
scn1lab	Number positive	1	1	3	5	0	0	0	0	0
	Number negative	29	29	29	29	29	29	29	29	29
	% positive	3	3	10	17	0	0	0	0	0
ube3a	Number positive	2	1	1	2	3	0	0	0	0
	Number negative	31	31	31	31	31	31	31	31	31
	% positive	6	3	3	6	10	0	0	0	0
Scrambled (plate B)	Number positive	1	3	2	1	0	0	1	0	0
	Number negative	34	34	34	34	34	34	35	34	34
	% positive	3	9	6	3	0	0	3	0	0
gabra1	Number positive	4	0	2	3	1	0	0	0	0
	Number negative	39	39	39	39	39	39	39	39	0
	% positive	10	0	5	8	3	0	0	0	0
pcdh19	Number positive	5	2	6	5	0	0	0	0	0
	Number negative	36	36	36	36	36	36	36	36	36
	% positive	14	6	17	14	0	0	0	0	0
Scrambled (plate C)	Number positive	1	0	0	0	1	0	0	0	0
	Number negative	37	37	37	37	37	37	37	37	37
	% positive	3	0	0	0	3	0	0	0	0
	Number	1	0	0	0	3	0	0	0	0

	positive									
kcnq2a (single)	Number negative	36	36	36	36	36	36	36	36	36
	% positive	3	0	0	0	8	0	0	0	0
kcnq2a (double)	Number positive	0	1	0	0	0	0	0	0	0
	Number negative	34	34	34	34	34	34	34	34	34
	% positive	0	3	0	0	0	0	0	0	0
kcnq2a/kcnq2b (single)	Number positive	3	1	1	0	3	0	0	0	0
	Number negative	35	35	35	35	35	35	35	35	35
	% positive	9	3	3	0	6	0	0	0	0
Scrambled (plate D)	Number Positive	1	0	0	0	0	0	0	0	0
	Number negative	25	25	25	25	25	25	25	25	25
	% positive	4	0	0	0	0	0	0	0	0
adgrg1	Number positive	6	6	1	4	2	0	0	0	0
	Number negative	29	29	29	29	29	29	29	29	29
	% positive	21	21	3	14	7	0	0	0	0

Supplementary Table S1. Analysis of different qualitative morphological alterations in the different gene-specific crispants.

5. Supplementary Table S2

		Heart area (μm2)		Eyes diameter (μm)		Body length (μm2)	
		**Value**	**Significance**	**Value**	**Significance**	**Value**	**Significance**
Scrambled (plate A)	Mean	41976	-	333.90	-	3690.0	-
	SEM	1964	-	3.68	-	39.8	-
	n	25	-	24	-	25	-
scn1lab	Mean	38954	n.s	337.10	n.s	3632.0	n.s
	SEM	1886	n.s	2.27	n.s	28.1	n.s
	n	29	n.s	27	n.s	29	n.s
ube3a	Mean	38986	n.s	340.90	n.s	3670.0	n.s
	SEM	1599	n.s	2.34	n.s	26.3	n.s
	n	30	n.s	31	n.s	31	n.s
Scrambled (plate B)	Mean	40353	-	341.20	-	3783.0	-
	SEM	2316	-	2.62	-	21.1	-
	n	34	-	32.00	-	34	-
gabra1	Mean	40339	n.s	339.60	n.s	3637.0	***
	SEM	1371	n.s	2.32	n.s	32.2	***
	n	38	n.s	39.00	n.s	39	***
pcdh19	Mean	42606	n.s	337.30	n.s	3566.0	***
	SEM	2508	n.s	2.68	n.s	31.4	***
	n	36	n.s	34.00	n.s	36	***
Scrambled (plate C)	Mean	32493	-	336.10	-	3563.0	-
	SEM	1026	-	1.59	-	32.5	-
	n	36	-	36.00	-	36	-
kcnq2a (single)	Mean	34161	n.s	343.30	*	3592.0	n.s
	SEM	1298	n.s	2.03	*	21.4	n.s
	n	36	n.s	36.00	*	36	n.s
kcnq2a (double)	Mean	33213	n.s	348.10	**	3703.0	***
	SEM	1128	n.s	2.42	**	23.8	***
	n	34	n.s	34.00	**	34	***
kcnq2a/ kcnq2b (single)	Mean	35498	n.s	337.90	n.s	3565.0	n.s
	SEM	1023	n.s	2.28	n.s	28.5	n.s
	n	34	n.s	35.00	n.s	35	n.s
Scrambled (plate D)	Mean	27766	-	337.00	-	3668.0	-
	SEM	514	-	2.03	-	23.3	-
	n	23	-	24.00	-	25	-
adgrg1	Mean	38523	***	304.00	***	3399.0	***
	SEM	4736	***	4.15	***	37.9	***
	n	27	***	29.00	***	29	***

Supplementary Table S2. Analysis of different quantitative morphological alterations in the different gene-specific crispants.

6. Supplementary Table S3

Variable	Description
Maximum speed (mm/s)	Maximum velocity reached by larvae in the two seconds after the light trigger in the trial
Maximum acceleration (mm/s^2)	Maximum acceleration reached by larvae in the two seconds after the light trigger in the trial
Mobility of the larvae in the area (%)	The total percentage change (in pixels) in the detected body area when compared with the previous measure after the light trigger in the trial
Mobility state	Definition of a discrete variable with three possible states, defined by different thresholds of movement. It is the calculation of the duration for whivh the complete area detected as animal is changing, even if the center point remains the same
Immobile (s)	Cumulative time after the light trigger in the trial when the larvae has been immobile, below the 20% of mobility threshold
Mobile (s)	Cumulative time after the light trigger in the trial when the larvae has been moving normaly, between 20% and 60% of mobility threshold
Highly mobile (s)	Cumulative time after the light trigger in the trial when the larvae has presented high mobility, above 60% of mobility threshold
Number of angle turns	Number of turns performed by larvae after the light trigger in the trial

Supplementary Table S3. Description of the different kinematic variables used for the PCA analysis.
